# Normative biometry of the fetal brain using magnetic resonance imaging

**DOI:** 10.1007/s00429-016-1342-6

**Published:** 2016-11-24

**Authors:** Vanessa Kyriakopoulou, Deniz Vatansever, Alice Davidson, Prachi Patkee, Samia Elkommos, Andrew Chew, Miriam Martinez-Biarge, Bibbi Hagberg, Mellisa Damodaram, Joanna Allsop, Matt Fox, Joseph V. Hajnal, Mary A. Rutherford

**Affiliations:** 1 Centre for the Developing Brain, Division of Imaging Sciences and Biomedical Engineering, Perinatal Imaging and Health, King’s College London, King’s Health Partners, St. Thomas’ Hospital, 1st Floor South Wing, London, SE1 7EH UK; 2 Gillberg Neuropsychiatry Centre, Institute of Neuroscience and Physiology, Sahlgrenska Academy at University of Gothenburg, Kungsgatan, 12 411 18 Gothenburg, Sweden

**Keywords:** Brain development, Magnetic resonance imaging, Centiles

## Abstract

The fetal brain shows accelerated growth in the latter half of gestation, and these changes can be captured by 2D and 3D biometry measurements. The aim of this study was to quantify brain growth in normal fetuses using Magnetic Resonance Imaging (MRI) and to produce reference biometry data and a freely available centile calculator (https://www.developingbrain.co.uk/fetalcentiles/). A total of 127 MRI examinations (1.5 T) of fetuses with a normal brain appearance (21–38 gestational weeks) were included in this study. 2D and 3D biometric parameters were measured from slice-to-volume reconstructed images, including 3D measurements of supratentorial brain tissue, lateral ventricles, cortex, cerebellum and extra-cerebral CSF and 2D measurements of brain biparietal diameter and fronto-occipital length, skull biparietal diameter and occipitofrontal diameter, head circumference, transverse cerebellar diameter, extra-cerebral CSF, ventricular atrial diameter, and vermis height, width, and area. Centiles were constructed for each measurement. All participants were invited for developmental follow-up. All 2D and 3D measurements, except for atrial diameter, showed a significant positive correlation with gestational age. There was a sex effect on left and total lateral ventricular volumes and the degree of ventricular asymmetry. The 5th, 50th, and 95th centiles and a centile calculator were produced. Developmental follow-up was available for 73.1% of cases [mean chronological age 27.4 (±10.2) months]. We present normative reference charts for fetal brain MRI biometry at 21–38 gestational weeks. Developing growth trajectories will aid in the better understanding of normal fetal brain growth and subsequently of deviations from typical development in high-risk pregnancies or following premature delivery.

**Electronic supplementary material:**

The online version of this article (doi:10.1007/s00429-016-1342-6) contains supplementary material, which is available to authorized users.

## Introduction

The latter half of gestation (20–40 gestational weeks) is a dynamic period during which the human brain shows accelerated growth manifesting in increases in volume, cortical complexity and changes in the molecular and cellular composition of the different brain regions. Magnetic resonance imaging (MRI) is a safe and complementary imaging modality, to ultrasound, to assess the fetal brain offering high-resolution and soft-tissue contrast. The extensive use of ultrasound as a screening modality has allowed for the production of biometric reference data in large fetal cohorts. While the use of MRI is becoming increasingly widespread, there is lack of a comprehensive high-quality MRI-based biometry data set of the normal fetus. While previous studies have measured brain parenchyma and volumes of intracranial structures, these have had several limitations (Clouchoux et al. 2011; Corbett-Detig et al. 2011; Gong et al. 1998; Grossman et al. 2006; Hu et al. 2009; Limperopoulos et al. 2010; Rajagopalan et al. 2011; Scott et al. 2011). Limitations have included the use of non-reconstructed data sets sensitive to fetal and maternal motion (Clouchoux et al. 2011; Gong et al. 1998; Grossman et al. 2006), small gestational age range (Corbett-Detig et al. 2011; Gholipour et al. 2011; Gong et al. 1998; Scott et al. 2011), limited sample size (Corbett-Detig et al. 2011; Gong et al. 1998; Grossman et al. 2006; Scott et al. 2011), and inclusion of clinical cases (Gholipour et al. 2011; Hu et al. 2009). The aims of this study were to quantify fetal brain development in normal fetuses using MRI and to produce a freely available online centile calculator.

## Methods

### Cohort

Ethical approval for the study was granted by the West London & GTAC Research Ethics Committee (Ethics No. 07/H0707/105), and written informed consent for participation was obtained from all pregnant participants. The participants for the normal control cohort comprised of healthy pregnant volunteers, women who had a previous child with a confirmed abnormality, or who had had a suspected fetal abnormality on ultrasound not present on MRI, or a mild non-CNS abnormality. Fetal brain MRI was performed at the Robert Steiner MRI Unit in Hammersmith Hospital between November 2007 and May 2013. Delivery summaries were obtained for all participants and reviewed for delivery complications and physical signs suggestive of genetic syndromes.

Participants with a singleton pregnancy with normal fetal brain appearance for gestational age, as reported on MRI, were included in the study. Subsequent exclusion criteria constituted delivery complications, congenital malformations or maternal infection, chromosomal abnormality, inadequate MR image quality, and abnormal developmental examination at either 1 or 2 years of age. Fetal gestational age (GA) was estimated from a first trimester dating ultrasound scan.

A total of 128 fetuses had a normal brain appearance on fetal MRI and were selected and evaluated for inclusion in the normal control cohort. 20 cases were excluded in accordance with our exclusion criteria: poor image quality due to fetal motion (1), positive infection screening (9), delivery complications (2), chromosome 22 duplication (1), language delay at 2 years of age (2), intrauterine growth restriction (1), seizures at 6 months of age (1), and no delivery summary and no parental contact after birth (3).

**Fig. 1:**
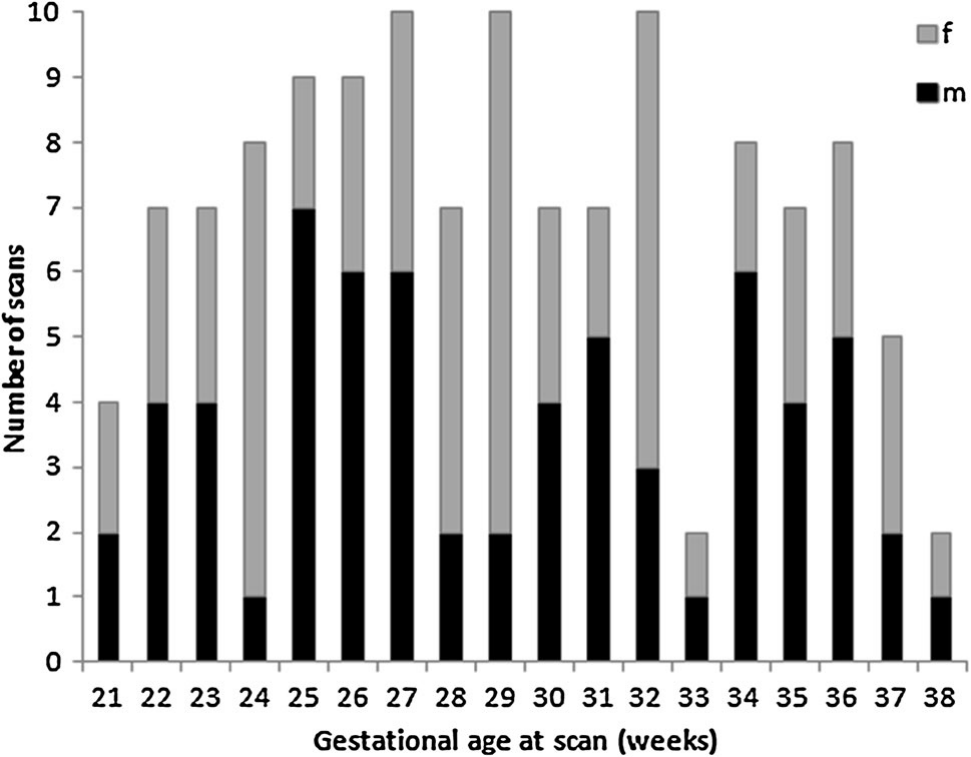
Gestational age and sex distribution in the normal control cohort. Histogram of gestational age and sex distribution of MR scans in normal fetuses. Fetal gestational age was estimated from a first trimester dating ultrasound scan. *f* female, *m* male

In the remaining 108 fetuses, MRI was performed at a median age of 29.43 week GA (range 21.29–38.86 weeks) (Fig. 1). 17 fetuses were scanned twice during gestation (range 27.86–38.71 weeks), while 1 fetus was scanned three times at different gestational ages. The normal control cohort consisted of 83 healthy volunteers, 15 women who had a previous child with a confirmed abnormality, 5 who had a suspected fetal abnormality on ultrasound excluded on MRI, and 5 who had a fetal mild non-CNS abnormality (Table 1). Clinical details regarding these cases are presented in detail in the Appendix. In summary, 127 MR scans of 108 fetuses were included in the control cohort (57 males/51 females).

**Table 1:** Cohort demographics

Cohort demographics	No. of fetuses	No. of scans	GA range (weeks)	Mean
Healthy volunteer	83	95	21.29–38.86	30.19
Sibling with disability	15	21	21.71–34.86	27.56
Ultrasound query CNS abnormality	5	6	23.14–37.71	27.14
Non-CNS mild abnormality	5	5	21.14–28.14	25.6
Total	108	127	21.29–38.86	29.43

### Imaging

#### Neuroimaging and reconstruction

Fetal MRI was performed using a 1.5 T MRI System (Philips Achieva; Philips Medical systems, Best, the Netherlands) with a 32-channel cardiac array coil placed around the mother’s abdomen. The mother was positioned in a left lateral tilt, no sedation was used, and the total duration of the MR examination did not exceed 60 min. Maternal temperature was measured using a tympanic thermometer prior to and after the scan. In instances, when the maternal temperature was ≥37.5 °C, the scan was rescheduled. A complete fetal brain clinical examination was performed in transverse, sagittal, and coronal planes. T2-weighted Single Shot Turbo Spin Echo (ssTSE) was acquired using the following scanning parameters: TR = 15,000 ms, TE = 160 ms, slice thickness of 2.5 mm, slice overlap of 1.5 mm, and flip angle = 90°. 3D reconstructed images were constructed using Snapshot MRI with Volume Reconstruction (SVR), as previously described (Jiang et al. 2007; Kuklisova-Murgasova et al. 2012). In summary, data sets from multiple ssTSE image stacks were acquired in three orthogonal planes using overlapping slices (four transverse, two coronal, and two sagittal acquisitions). The fetal brain was oversampled to ensure the acquisition of complete data sets even with significant motion. Post-acquisition processing and registration of raw images was performed on Windows and Linux workstations (total duration 40 min). All scans were reviewed for image quality, and the slices corrupted by motion artefacts and loss of anatomical detail were excluded from the proceeding analysis. Image registration is performed to align all images obtained based on the assumption of a rigid body, of constant shape and size, performing an unknown motion. Images were registered onto a self-consistent anatomical space of the fetal brain (volume with least motion), and using a scattered interpolation approach, all measured voxel intensities are used to reconstruct the 3D fetal brain with an accuracy of 0.3 mm. The reconstructed 3D volumetric data sets have high resolution, high signal-to-noise ratio, and full brain coverage essential for reliable volumetric analysis. Visual analysis of all acquired images was performed by an expert radiologist to exclude additional anomalies and confirm appropriate appearance for gestation. The 3D fetal volumetric brain data were orientated into standard axial, coronal, and sagittal projections, and the voxel size was interpolated from a reconstruction voxel size of 1.18 ×  1.18 ×  1.18 mm to 0.2  ×  0.2  ×  1 mm to aid visual display and assist placement of anatomical markers.

#### Quantification analysis (3D)

**Fig. 2:**
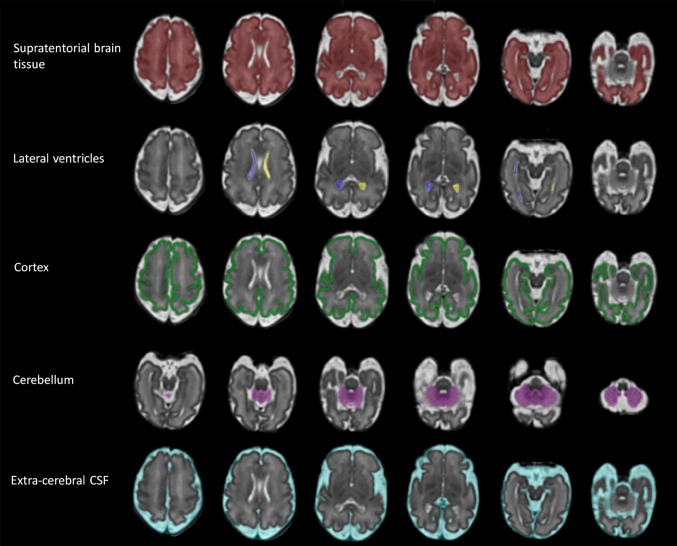
3D measurements: guidelines. 3D reconstructed brain of a normal control fetus at 32 gestational weeks with superimposed 3D segmentations of supratentorial brain tissue, lateral ventricles, cortex, cerebellum, and extra-cerebral CSF

Volumetric measurements were produced on the 3D reconstructions from semi-automatic segmentations using ITK-SNAP (version 2.2.0, University of Pennsylvania, Philadelphia, PA, USA) (Yushkevich et al. 2006) in a two-step process. Automatic segmentation of the different intracranial regions is based on image contrast while utilising user-defined thresholds. Following completion of the automatic process, editing of each segmentation was performed manually using a digital drawing tablet (Intuos, Wacom, Germany) to remove incorrectly labelled areas. Supratentorial brain tissue volume was defined as the brain tissue above the tentorium, i.e., excluding the brainstem, cerebellum, and cerebrospinal fluid (CSF) spaces (Fig. 2). Total ventricular volume was defined as the volume of both left and right lateral ventricles including the choroid plexus but excluding the third and fourth ventricles and cavum septum pellucidum and vergae (CSP) (Fig. 2). Lateral ventricle volume refers to the volume of each lateral ventricle. Laterality was established by the position of the fetal heart, stomach, and liver on MR images in a coronal plane (no fetuses had situs inversus or dextrocardia as assessed on antenatal ultrasound). Cortical volume represents the total cerebral cortical gray matter and was manually segmented in 75 scans (37 males/38 females and GA range 21.29–38.86) (Fig. 2). Cortical segmentation was only performed in a sub-group of our cohort (chosen to span gestation) due to the laborious nature of the current segmentation process (~6 h per segmentation). The total cerebellar volume measurement included both the cerebellar and the vermis volumes and excluded the fourth ventricle (Fig. 2). Extra-cerebral CSF included all intracranial CSF spaces surrounding the supratentorial brain tissue and cerebellum and including the interhemispheric fissure space but not any ventricular structure or the CSP (Fig. 2). The time required for the manual editing of the different structures varied and were as following for a 28-week-old fetus: supratentorial brain tissue (1 h), total lateral ventricles (10 min), cortex (6 h), cerebellum (15 min), and extra-cerebral CSF (30 min).

#### Quantification analysis (2D)

**Fig. 3:**
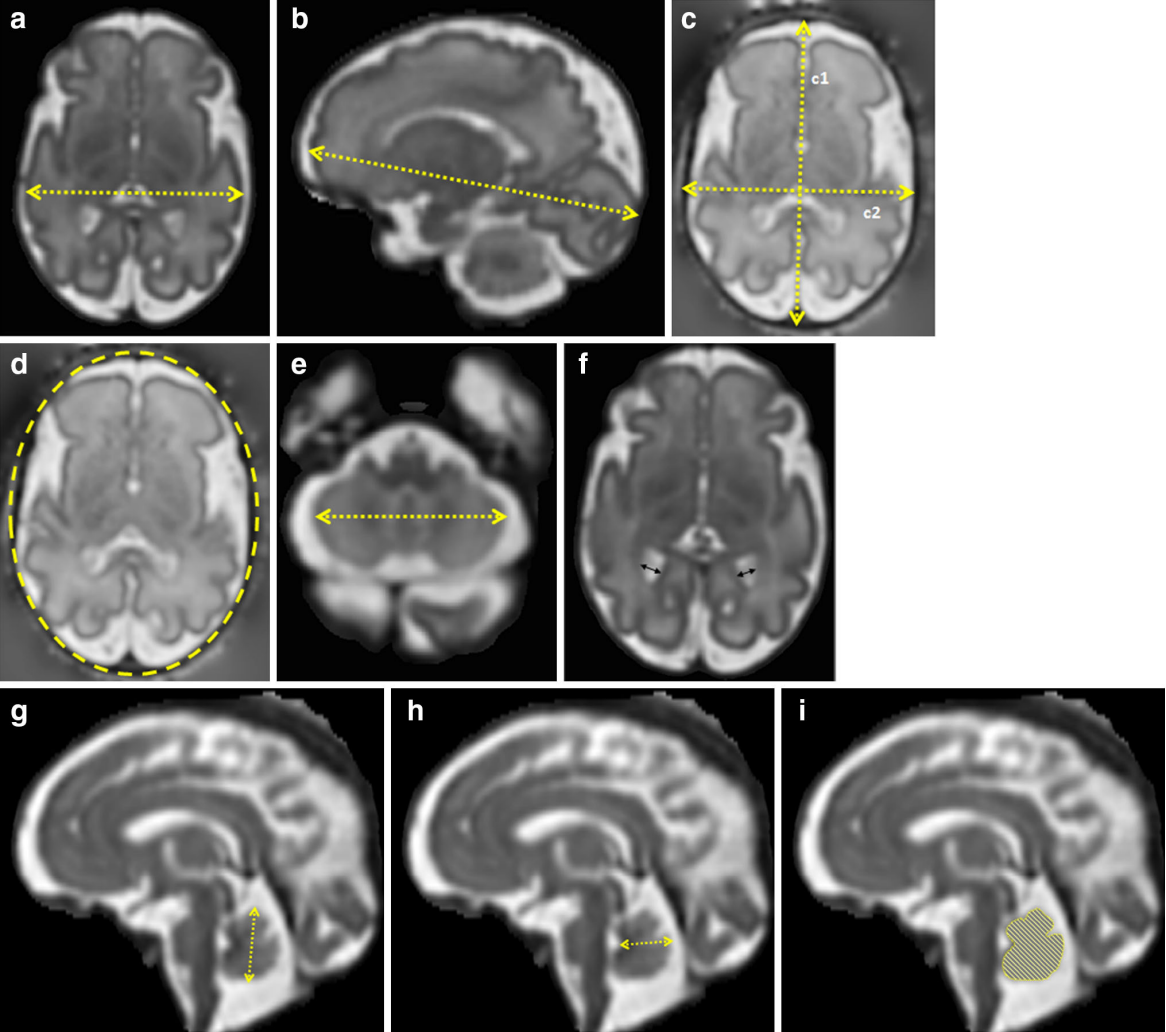
2D measurements: guidelines. **a** Brain biparietal diameter, **b** brain fronto-occipital length, **c** skull occipitofrontal diameter (*c1*) and skull biparietal diameter (*c2*), **d** head circumference using the eclipse tool, **e** transverse cerebellar diameter, **f** atrial diameter, **g** vermis height, **h** vermis width, and **i** vermis area

Linear measurements were performed on the 3D reconstructions using ImageJ (version 1.40 g, National Institutes of Health, Bethesda, MD, USA) and included the brain biparietal diameter and fronto-occipital length, skull occipitofrontal diameter and biparietal diameter, head circumference, transverse cerebellar diameter, extra-cerebral CSF, atrial diameter, and vermis height, width, and area. The brain biparietal diameter was measured in a transverse plane as the maximum brain width (Fig. 3a). The fronto-occipital length of each hemisphere was measured in a sagittal plane as the distance between the extreme point of the frontal and occipital lobes (Fig. 3b). The skull occipitofrontal diameter was defined as the maximum distance between the frontal and occipital skull bones and was measured in a transverse plane by placing the cursors in the middle of the bone hypo-intense area (Fig. 3c1). The skull biparietal diameter was defined as the widest diameter of the fetal skull measured in a transverse plane using the “outer edge to inner edge” technique (Fig. 3c2) (Salomon et al. 2010). The head circumference was measured in two different ways to reflect the different measuring techniques used in ultrasonography. First using the equation: head circumference = 1.62 × [(skull biparietal diameter) + (skull occipitofrontal diameter)] and second using the eclipse tool, option in ImageJ, surrounding the fetal skull (Fig. 3d) (Salomon et al. 2010). The transverse cerebellar diameter was defined as the maximum lateral cerebellar distance in the transverse plane (Fig. 3e). The linear measurement of extra-cerebral CSF was calculated using the following formula: (skull biparietal diameter) − (brain biparietal diameter). The atrial diameter was measured according to the guidelines of the International Society of Ultrasound in Obstetrics and Gynaecology (ISUOG 2007) at the level of the atrium (Fig. 3f). More specifically on MRI, the atrial diameter was measured on a slice, where both the posterior aspects of the basal ganglia and the third ventricle were visible. The cursors are placed inside the low signal intensity of the inner edge of the ventricular wall and perpendicular to the long axis of the ventricle. The vermis height, width, and area were measured in the mid-sagittal plane (Fig. 3g–i). The vermis height corresponded to the maximum superior–inferior length and the width to the maximum distance between the fastigium and the posterior part of the vermis in the mid-sagittal plane. The vermis area was calculated using a free-hand drawing tool.

### Developmental assessments

All children were invited for a developmental assessment at 1 and 2 years of age to ensure that normative data produced represent typically developing children. Assessments were performed by a clinical psychologist or paediatric neurologist. The Griffiths Mental Development Scale (GMDS) assessment was performed at year 1, and the Bayley-III Scales of Infant and Toddler Development (Bayley-III) assessment were performed at year 2 (Bayley 2006; Huntley 1995). The GMDS is composed of five separate scales: locomotor, personal–social, hearing and speech, eye-hand co-ordination, and performance. Developmental Quotient (DQ) scores in the range of 88-112 and Sub-Quotient (SQ) scores of 84–116 were considered to represent typical development. Developmental delay was defined as a DQ score below 88 (<1 standard deviation) (SD) or an SQ score below 84 (<1SD). The Bayley-III assessment comprises of three separate scales, cognitive, language (expressive and receptive), and motor (gross and fine). Scaled scores of 7–13 and composite scores of 85–115 were considered within typical development. Developmental delay was defined as a scaled score below 7 (<1SD) or a composite score below 85 (<1SD). Children scoring within the developmental delay range at any time point where excluded from the cohort. Parents that were unable to attend a developmental assessment were sent a parental filled questionnaire [Age and Stage Questionnaires–III (ASQ-III)] assessing communication, gross motor, fine motor, problem solving, and personal–social skills (age range 1–66 months). ASQ-III is a set of age-specific questionnaires (age range 1–66 months) that serve as a developmental screening modality. Developmental delay was defined as a score <2SD and children scoring within the developmental delay range at any timepoint where excluded from the cohort.

### Statistical analysis

Statistical analysis was performed using the SPSS software package version 17 (SPSS Chicago, IL, USA). Normality of distribution was assessed using the Shapiro–Wilk goodness-of-fit test and the Q–Q plots for each variable. Correlation between variables was assessed with the Spearman’s rank correlation coefficient (*r*). A confidence level of 0.05 was considered significant. The Bonferroni adjustment was applied. Multiple raters were involved in the study. All raters received extensive training to obtain reliability and achieve a percentage difference between 2D and 3D measurements of less than 5%. Intra- and inter-rater reliability was performed for all measurements and included image spanning the GA range of the cohort. Intra-rater and inter-rater variability was assessed using the intra-class correlation coefficient and Bland–Altman plots on SPSS. The intra- and inter-class correlation coefficients for all 2D and 3D measurements were 0.99 (*p* < 0.0001). The relative growth rate represents the percent volume gain relative to the average volume for each structure. This was calculated assuming linear growth to make our results comparable to previous studies. The relative growth rate represents the percent volume gain relative to the average volume for each structure and was calculated using the formula: Relative Growth Rate = [(ln*V*_2_-ln*V*_1_)/(GA_2_ − GA_1_)] × 100, where ln is the natural logarithm, GA_1_ and GA_2_ are the gestational weeks at a given GA range, and *V*_1_ and *V*_2_ are the volumes of the intracranial structure at timepoints GA_1_ and GA_2,_ respectively (Hoffmann and Poorter 2002). Slope comparison was performed in the StatsDirect statistical software (version 3.0) using linear regression.

The 5th, 50^th^, and 95th centiles for the 2D and 3D measures of each intracranial structure were constructed, as described by Royston and Wright (1998). This approach is based on a linear regression that models both the mean and SD across GA. Briefly, least-square regression analysis was used to estimate the mean curves of each measurement as polynomial functions of GA. A quadratic line showed the best fit for all 2D and 3D measurements, except for cortical volume, where an exponential fit was representative. The scaled residuals were calculated, and polynomial regression analysis was performed to estimate an appropriate curve representing the SD. A straight line was adequate for the SD of all 2D and 3D measurements, except for the cortical volume SD, where a quadratic line was more appropriate. The 5th, 50th and 95th centiles were calculated using the equation: centiles = mean + K × SD, where the mean and SD were substituted by the appropriate curve as estimated above and K is the corresponding centiles of the standard Gaussian distribution.

### Agreement analysis of 2D measurements performed prior and after SVR reconstruction

The SVR methodology ("Neuroimaging and reconstruction") may not be readily available in clinical environments, and 2D measurements will often be performed on non-reconstructed images. To ensure reliable use of the calculator using 2D measurements performed prior and after SVR reconstruction, we have performed statistical agreement analysis between 2D measurements performed on T2-weighted ssTSE (non-reconstructed images typically acquired in a clinical scan) and their corresponding reconstructed images. All 2D measurements were performed by the same rater. The analysis was performed in a sub-cohort of ten fetuses (GA 21.71, 22.86, 25.29, 26.14, 28.29, 29.57, 31.71, 32.71, 34, and 36 weeks). Cases were selected to span the gestational period studied and only included symmetrical non-rotated images.

## Results

Normative biometric indices for 2D and 3D measurements were generated from our cohort of 127 brain MR images ranging from 21.29 to 38.86 gestational weeks. The 5th, 50th, and 95th centiles were created for all 2D and 3D measurements.

## 3D volumetric measurements

The results are presented in absolute volumes, and relative and average growth rates and are summarised in Table 2. All volumetric measurements had significant positive correlations with GA and a quadratic line was depicted as the best fit model for each structure, except for the cortical volume for which an exponential fit was most appropriate.

**Table 2:** 3D measurement fetal brain biometry

	Supratentorial brain tissue	Total lateral ventricles	Cortex	Cerebellum	Extra-cerebral CSF
Spearman’s r	0.98**	0.483**	0.967**	0.986**	0.856**
Average volume (cm^3^)					
At 22 GW	43.26	2.51	10.19	1.57	26.90
At 26 GW	92.26	3.29	17.38	3.77	60.85
At 30 GW	153.31	3.99	29.63	7.38	84.43
At 34 GW	226.39	4.60	50.51	12.38	97.63
At 38 GW	311.52	5.14	86.12	18.78	100.45
Relative growth rate (%/week)	13.04	3.86	14.78	17.31	9.60
Absolute growth rate (cm^3^/week)					
At 22 GW	10.75	0.20	1.36	0.38	9.78
At 26 GW	13.76	0.18	2.32	0.73	7.19
At 30 GW	16.77	0.16	3.95	1.08	4.60
At 34 GW	19.78	0.14	6.74	1.43	2.00
At 38 GW	22.79	0.12	11.49	1.78	−0.59

Volumetric results for normal control cohort. The correlation of the volume of each intracranial structure and GA is indicated by the Spearman’s *r* value. Relative growth rate represents the percent volume increase relative of the average volume of the structure. ** *p* < 0.0001

**Fig. 4:**
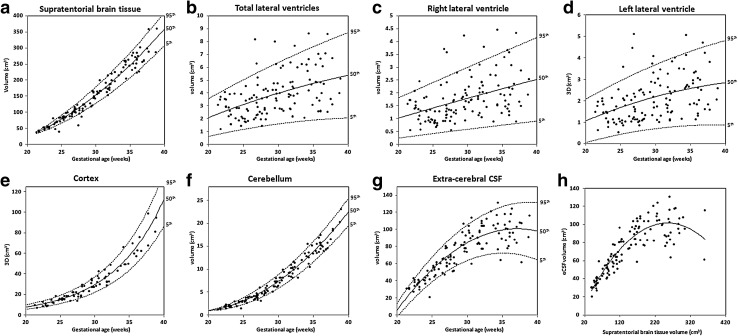
3D measurements: growth trajectories and centiles. Best fit models for normal control 3D growth trajectories of **a** supratentorial brain tissue, **b**–**d** total and left and right lateral ventricles, **e** cortex, **f** cerebellum, and **g** extra-cerebral CSF. *Solid lines* depict the 50th centile, and *dotted lines* the 5th and 95th centiles. **h** Correlation of extra-cerebral CSF and supratentorial brain tissue volumes, *eCSF* extra-cerebral CSF

Total supratentorial brain tissue volume increased at a relative growth rate of 13.04% per week with increasing GA, from 43.26 cm^3^ at week 22 to 311.52 cm^3^ at week 38 (Spearman’s *r* = 0.98, *p* < 0.0001) (Fig. 4a; Table 2).

The lateral ventricles were assessed using the volume of each lateral ventricle and then the total ventricular volume. There was a small increase in the ventricular size with increasing GA as indicated by a moderate correlation value in the volume of each lateral ventricle (left: Spearman’s *r* = 0.464 and right: *r* = 0.445, *p* < 0.0001) and total lateral ventricular volume (Spearman’s *r* = 0.483, *p* < 0.0001) with GA (Fig. 4b–d). The total lateral ventricular volume increased at a relative rate of 3.86% per gestational week increasing from an average volume of 2.51 cm^3^ at week 22 to 5.14 cm^3^ at week 38 (Table 2). Increased inter-subject variation, however, was evident in all ventricular measurements. The total ventricular-to-brain volume ratio depicted a deceleration in ventricular growth also confirmed by the relative growth rates of 3.86% for the total lateral ventricles and 13.04% for the supratentorial brain tissue.

Cortical volume was measured in 75 control cases (GA range 21.29–38.86 weeks, mean 29.22 weeks). Cortical volume increased at a relative growth rate of 14.78% with increasing GA, from 10.19 cm^3^ at week 22 to 86.12 cm^3^ at week 38 (Spearman’s *r* = 0.967, *p* < 0.0001) (Fig. 4e; Table 2). Cortical volume increased at a significantly higher rate compared to supratentorial brain volume (p < 0.0001).

Total cerebellar volume increased at a relative growth rate of 17.31% with increasing GA, from 1.57 cm^3^ at week 22 to 18.78 cm^3^ at week 38 (Spearman’s *r* = 0.986, *p* < 0.0001) (Fig. 4f; Table 2). Cerebellar volume increased at a significantly higher rate compared to supratentorial brain volume (*p* < 0.0001).

Extra-cerebral CSF increased with GA at a rate of 9.6% per week from 26.9 cm^3^ at week 22 to 100.45 cm^3^ at week 38 (Spearman’s *r* = 0.856, *p* < 0.0001) (Fig. 4g; Table 2). There was a large scatter of data from 30 gestational weeks which may indicate a decrease in the rate of change during this period. Extra-cerebral CSF correlated strongly with supratentorial brain tissue from 22 to 30 weeks (Spearman’s *r* = 0.894, *p* < 0.0001) and weakly from 30 to 38 weeks (Spearman’s *r* = 0.268, *p* = 0.035) (Fig. 4h).

## 2D linear measurements

**Fig. 5:**
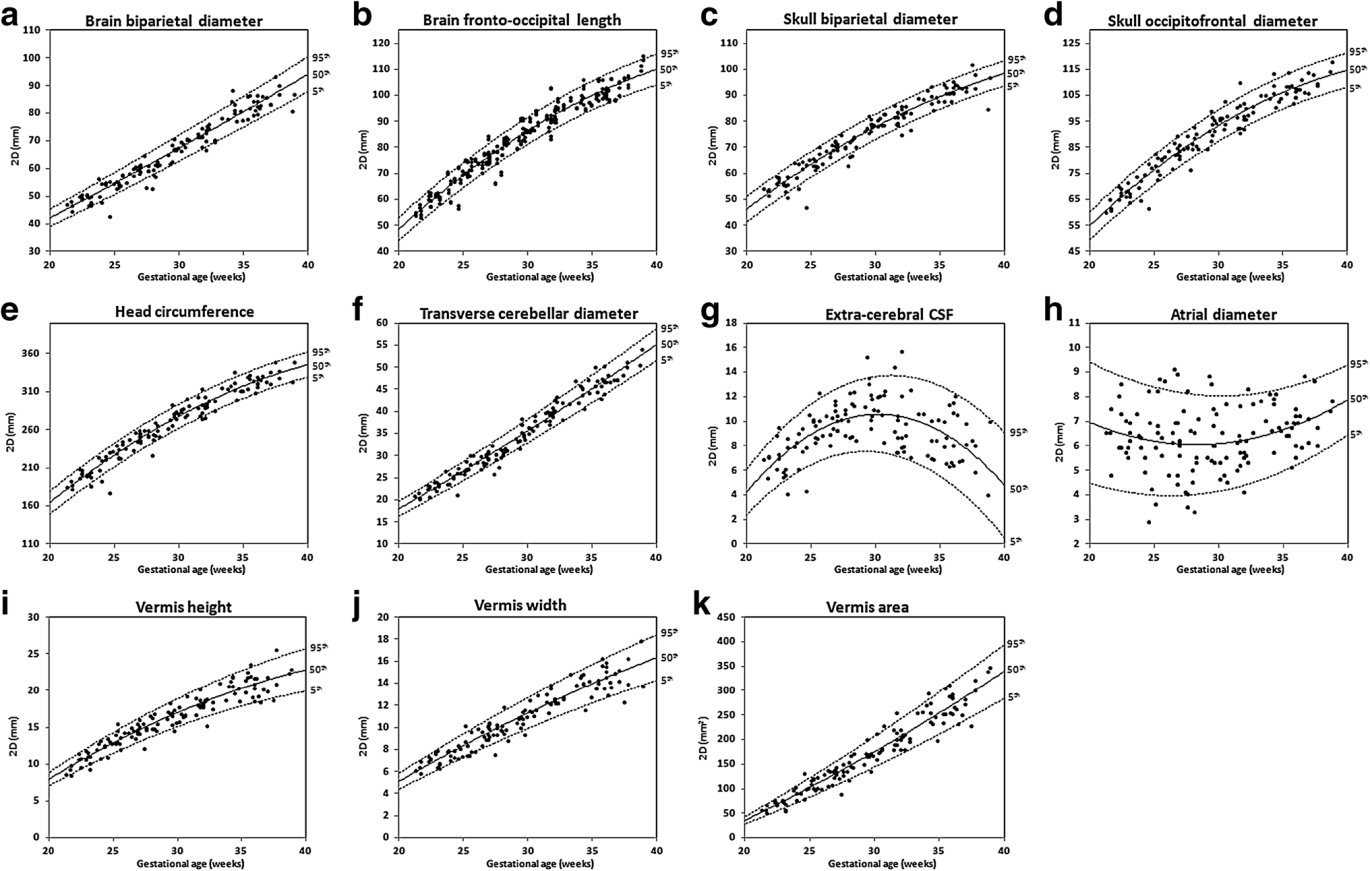
2D measurements: growth trajectories and centiles. Best fit models for normal control 2D growth trajectories of **a** brain biparietal diameter, **b** brain fronto-occipital length, **c** skull biparietal diameter, **d** skull occipitofrontal diameter, **e** head circumference, **f** transverse cerebellar diameter, **g** extra-cerebral CSF, **h** atrial diameter, **i** vermis height, **j** vermis width, and **k** vermis area. *Solid lines* depict the 50th centile, and *dotted lines* the 5th and 95th centiles

2D measurements and their growth trajectories and centiles are presented in Fig. 5a–k. The 2D measurements of the brain biparietal diameter (*r* = 0.968, *p* < 0.0001), brain fronto-occipital length (*r* = 0.973, *p* < 0.0001), skull occipitofrontal diameter (*r* = 0.965, *p* < 0.0001) and skull biparietal diameter (*r* = 0.966, *p* < 0.0001), head circumference (*r* = 0.972, *p* < 0.0001), transverse cerebellar diameter (*r* = 0.982, *p* < 0.0001), vermis height (*r* = 0.95, *p* < 0.0001), vermis width (*r* = 0.949, *p* < 0.0001), and vermis area (*r* = 0.964, *p* < 0.0001) showed strong positive correlations with GA. There was no significant correlation with GA for the left and right atrial diameter (Spearman’s *r* = 0.118, *p* = 0.091, and *r* = 0.082 *p* = 0.179, respectively). The mean atrial diameter in our cohort was 5.97 ± 1.42 mm. There was no significant difference between the left and right hemisphere fronto-occipital lengths in the cohort (*p* = 0.619); therefore, these are presented together. There was no significant difference between the two techniques for measuring head circumference (*p* = 0.318). We have presented the data for the head circumference measured using the formula head circumference = 1.62 × [(skull biparietal diameter) + (skull occipitofrontal diameter)] as the eclipse tool measurement is not an option readily available on MR workstations. The calculated linear measurement of extra-cerebral CSF = (skull biparietal diameter) − (brain biparietal diameter) showed an increase from 22 to 30 weeks when it reached a peak and decreased from 30 to 38 weeks.

### Correlation between 2D and 3D measurements

There was a strong correlation between head circumference and the respective volumetric measurement (supratentorial brain + total lateral ventricles + extra-cerebral CSF) (*r* = 0.981, *p* < 0.0001) and between transverse cerebellar diameter and cerebellar volume (*r* = 0.986, *p* < 0.0001). Supratentorial brain tissue volume correlated strongly with head circumference (Spearman’s *r* = 0.985, *p* < 0.001). There was a moderate significant correlation between atrial diameter and lateral ventricle (*r* = 0.619, *p* < 0.0001) volume and for extra-cerebral CSF volume and its respective 2D measurement (skull biparietal diameter − brain biparietal diameter) (*r* = 0.439, *p* < 0.0001).

### Effect of sex

There was no significant sex effect for supratentorial brain tissue (*p* = 0.063), right ventricular (*p* = 0.075), cerebellar (*p* = 0.93), extra-cerebral CSF (*p* = 0.306), and cortical (*p* = 0.493) volumes. However, male fetuses had significantly larger total (*p* = 0.004) and left (*p* = 0.004) ventricular volumes compared to females. There was a significant difference of 17.91% between the left and right ventricular volumes (*p* = 0.001) in the entire cohort with the left side being larger. The left ventricle was significantly larger compared to the right in both males (difference = 22.02%, *p* = 0.003) and females (difference = 13.56%, *p* = 0.051). There was no correlation between the percentage of ventricular asymmetry and GA (Spearman’s *r* = −0.072, *p* = 0.418). There was no significant difference between sexes in any of the 2D measurements except for the transverse cerebellar diameter (*p* = 0.024), with males having larger measurements by 0.6 mm compared to females.

### Results of agreement analysis of 2D measurements performed prior and after SVR reconstruction

As previously described, agreement analysis was performed for all 2D measurements prior and after reconstruction in a sub-cohort of fetuses. The calculated intra-class correlation coefficient values were within the range of 0.95–0.99 (*p* < 0.001–*p* < 0.0001). The percentage error for the 2D measurements performed on T2-weighted ssTSE, and their corresponding reconstructed images was within the range of 0.2–2.4%, corrected for variability from intra-observer error.

### Follow-up

The mean GA at birth was 39.5 weeks (range 36–42.4 weeks), and the mean birthweight was 3.248 kg (range 2.16–4.42 kg). The median Apgar score was 9 (range 5–10) at 1 min and 10 (range 8–10) at 5 min. All the children enrolled in the study were invited for a developmental assessment at year 1 and year 2 of age. Follow-up (GMDS or BSID-III or ASQ-III) was available in 81 children representing a 75% follow-up rate at a mean chronological age of 27.4 (± 10.2) months and a range of 11.7–63 months. 52 children had a GMDS assessment at a mean chronological age of 13.5 (±1.9) months (range 8.5–18.8 months) and received scores within the normal range. 33 children had a BSID-III assessment at a mean chronological age of 25.9 (±1.8) months (range of 20.1–29.4 months). two children received scores below 1SD in the language subscale and were, therefore, removed from the cohort. The remaining 31 children received scores within the normal range. 13 children completed an ASQ-III and did not attend a formal assessment [mean 34.1 (±12.7) months, range 20–63 months]. In summary, out of the 108 children participating in the study, 79 children (73.1% follow-up rate) received follow-up and scored within the typical developing range, while 29 children did not have any follow-up. There was no significant difference between the cohorts with and without follow-up in 2D or 3D measures, GA at scan, GA at birth, birthweight corrected for GA at birth, or Apgar scores. We have not performed any correlations between MR measures and developmental scores as this is part of a separate study.

## Discussion

This is the largest study to date, to the best of our knowledge, to produce 2D and 3D MRI biometric reference data using 127 fetal brain images from normal fetuses ranging from 21.29 to 38.86 gestational weeks. Our normal control cohort has been carefully selected using strict inclusion criteria, and all children were enrolled in a follow-up program. In addition, we have created centile reference charts for 2D and 3D measurements for cerebral MRI biometry and a centile calculator.

## 3D volumetric measurements

The relative growth rates varied between structures with the cerebellum showing the fastest growth per week followed by the supratentorial brain tissue and the cortex, while the growth of the lateral ventricles was the slowest. The supratentorial brain tissue followed a quadratic growth pattern indicating accelerated growth at this stage of development as demonstrated by the increasing growth velocity in brain weight in the third trimester (Guihard-Costa and Larroche 1992). The absolute volume increased between 22 and 38 weeks at a relative growth rate of 13.04% per week. This is comparable with previous MR volumetric studies, performed on 3D reconstructed data within smaller gestational age ranges, which presented growth ranges of 10.22–17% (Clouchoux et al. 2011; Rajagopalan et al. 2011; Scott et al. 2011). The volume range increased from around 30 weeks of gestation suggesting that individual variation starts to arise and becomes greater with progressing age as previously shown (Guihard-Costa and Larroche 1992; Scott et al. 2011; Snijders and Nicolaides 1994). Cortical volume increased at a relative growth rate of 14.78% per week in our sub-cohort of fetuses. This is in agreement with a previous study performed on 34 normal fetuses at 20–31 gestational weeks (Scott et al. 2011). Cortical volume increased at a higher rate than the supratentorial brain tissue as demonstrated in the fetal (Scott et al. 2011) and preterm brain (Kapellou et al. 2006). Cortical volume was related to supratentorial brain tissue volume by a scaling exponent of 1.1. Our absolute cerebellar volumes are in agreement with earlier MR studies (Clouchoux et al. 2011; Grossman et al. 2006; Hatab et al. 2008; Limperopoulos et al. 2010). The relative growth rate of the cerebellum exceeded that of the supratentorial brain tissue as previously shown in post-mortem and MR studies (Clouchoux et al. 2011; Grossman et al. 2006; Hatab et al. 2008; Limperopoulos et al. 2010; Vatansever et al. 2013). The cerebellum exhibits an abrupt acceleration in growth velocity from 24 weeks of gestation in comparison with the supratentorial brain and maintains this acceleration until birth (Guihard-Costa and Larroche 1992; Moss and Noback 1956). Cerebellar volume related to supratentorial brain tissue volume by a scaling exponent of 1.25. The volume of the total lateral ventricles showed a moderate increase with increasing GA. This increase is minimal in comparison with brain growth. The existing MR volumetric studies have produced variable results as to whether the total volume of the lateral ventricles remains stable (Kazan-Tannus et al. 2007), increases (Scott et al. 2011) or decreases with GA (Clouchoux et al. 2011). This variability may be attributed to differences in the anatomical definition of the structure and inclusion of the third ventricle or the cavum septum pellucidum. We have previously shown in a sub-group (*n* = 60) of this cohort that the volumes of the CSP, third and fourth ventricles, change significantly with GA (Kyriakopoulou et al. 2014). Extra-cerebral CSF volume increased at a relative growth rate of 9.6% per week from 22 to 38 weeks. The volume of the extra-cerebral CSF had a strong correlation with supratentorial brain growth from 22 to 30 weeks and a weak correlation thereafter. Extra-cerebral CSF volume related to supratentorial brain tissue volume by a scaling law exponent of 0.6. In contrast to all other brain structures, extra-cerebral CSF showed a deceleration in growth from around 30 weeks of gestation. This is in agreement to the visual observation of the MR images and the 2D measures of skull biparietal diameter—brain biparietal diameter. Ultrasound data from normal fetuses indicate a decrease in peri-cerebral spaces during gestation (Garel 2004). Unfortunately, it is not possible to compare with other MR volumetric studies as the CSF measures in these include all intracranial CSF spaces.

Similar to the evolution pattern of the extra-cerebral CSF volume, the 2D and 3D measures of the CSP also peak at 30 weeks (Kyriakopoulou et al. 2014), while amniotic fluid volume follows a similar bell-shaped trajectory peaking at around 30–32 weeks (Brace and Wolf 1989). In addition, our data on intracranial parameters indicate an increase in volume range in the supratentorial brain tissue, cerebellum, total lateral ventricles, and cortex around that time. It may be speculated that 30 weeks of gestation is a significant timepoint of fetal brain development with various intracranial structures exhibiting changes in their growth trajectories.

### Correlation between 2D and 3D measurements

While all 2D measurements showed a strong positive correlation with increasing GA, neither the left nor right atrial diameter correlated with GA. Previous ultrasound studies have shown a significant but weak correlation with GA and the authors commented that this was not clinically significant (Almog et al. 2003; Hilpert et al. 1995). It is generally accepted in the literature that the ventricular atrium is relatively stable from 14 weeks of gestation until birth. Atrial diameter showed a moderate correlation with ventricle volume which we expected due to the complexity of the structure and variations that exist outside the posterior ventricular region. Similarly, extra-cerebral CSF had a moderate correlation with its respective 2D measurement (skull biparietal diameter–brain biparietal diameter) indicating that this linear measurement may not be sensitive enough to depict variation in extra-cerebral CSF volume. The extra-cerebral CSF linear measurement showed an increase from 22 to 30 weeks when it reached a peak and decreased from 30 to 38 weeks. Interestingly, this is a similar evolution pattern of the 2D and 3D measurements of the CSP (Kyriakopoulou et al. 2014). The 2D extra-cerebral CSF measurement will be predominantly affected by the development of the temporal lobes, while the 3D extra-cerebral CSF measurement will be affected by the development of all brain lobes. Therefore, we speculate that a difference in the trajectory of the 2D and 3D measures may be due to the differential growth rate of the brain lobes.

There was a strong correlation between head circumference and supratentorial brain tissue indicating that head circumference is an appropriate measure of brain size in appropriately developing fetuses. We measured the head circumference using two different methods as these are commonly used on antenatal ultrasound. Our data on head measurements are of great value for comparison with babies that have been born preterm and also with fetuses with a breech presentation as these are high-risk cohorts for developing biparietal head flattening.

### The effect of sex

Males had significantly larger left and total lateral ventricular volumes compared to females, and there was a non-significant trend for larger supratentorial brain tissue and right lateral ventricular volumes. In addition, ventricular asymmetry was greater in males compared to females (22.02 vs 13.56%, respectively). A small difference of 0.6 mm in transverse cerebellar diameter measures was identified between sexes and is unlikely to be clinically significant. Ultrasound studies have indicated a sex effect on the growth of various biometric parameters, such as atrial diameter and biparietal diameter; however, these are of small magnitude (Bromley et al. 1993; Nadel and Benacerraf 1995; Smulian et al. 1995). Gilmore et al. (2007) showed that male neonates had larger intracranial volumes to females.

## Strengths and limitations

The strengths of our study include a large gestational age range, sufficient number of scans per gestational week, registered volumetric data sets suitable for accurate volumetric analysis, rigid inclusion/exclusion criteria of participants, and comprehensive follow-up of the children to ensure typical development. Nevertheless, there are a few limitations to the current study. Cortical volume segmentations were performed only in a subset of fetuses due to the extensive work required to perform detailed and accurate manual segmentations. While automatic segmentation techniques are available, they still require substantial manual editing. In addition, we did not quantify different tissue types, such as white matter and central gray matter, although we have published their volumetric results previously in a small sub-cohort of this population (Kyriakopoulou et al. 2014). Our future plans include the use of these accurate segmentations as prior knowledge for the design of the automatic volumetric segmentation software. This would reduce the time required for manual editing following automatic segmentation. We were unable to follow the development of all our children in the cohort; however, we did not detect any differences (in 2D or 3D measures, GA at scan, GA at birth, and birthweight corrected for GA at birth or Apgar scores) between the children that had an assessment and those that were lost in follow-up. We have not performed a comparison or correlation in biometric measurements between ultrasound and MRI as this is beyond the scope of our study and will be addressed in a future study.

## Centile calculator

The centile calculator can be downloaded for free from https://www.developingbrain.co.uk/fetalcentiles/.

The calculator has been designed to allow for accurate centile calculation and automatic graph production. The calculator is in excel format and user friendly. First, it requires the input of the expected date of delivery (as calculated by a dating antenatal ultrasound scan) and the date of scan to automatically produce the GA at scan. 2D and 3D measurements can then be inserted into the input fields to generate the exact centiles for each measurement. Corresponding graphs for each measurement are produced depicting the 5th, 50th, and 95th centiles lines, the raw data from our normal control population and the location of the measurement inserted. The calculator has been formatted for printing.

Due to the fact that the SVR methodology may not be readily available in clinical settings, we performed an agreement analysis between 2D measurements done prior and after the SVR reconstruction. The small error (0.2–2.4%) suggested that 2D measurements can be reliably performed on T2-weighted ssTSE images, providing that images have been acquired in orthogonal planes.

In our department, image registration and segmentation of intracranial structures is an in-house process which aids clinical reporting. The use of the calculator for the production of centiles from 2D measurements can be achieved without image registration and, therefore, can be used in any clinical setting. The production of centiles from 3D measures requires image registration and segmentation of intracranial structures. This would require the appropriate acquisition of T2-weighted ssTSE images and a trained individual to perform image registration and structural segmentations using a pre-designed pipeline. As an example, the entire process from image registration to the production of a segmentation of the lateral ventricles and supratentorial brain tissue would take approximately 2 h. When the registration process was initially developed, the total duration of the pipeline process was 24–48 h (Jiang et al. 2007). In the future, we hope to optimise the process further with the eventual aim to provide real-time registration and fully automatic segmentation, thus allowing prompt reporting of both 2D and 3D image data sets.

Construction of quantitative normative trajectories of fetal intracranial structures is essential to understand the timing and progression of aberrant brain development and the early detection of deviations from normal growth during this period whether these occur inside or outside the uterine environment.

### Electronic supplementary material

Below is the link to the electronic supplementary material.

Supplementary material 1 (XLSX 4172 kb)

## Appendix

All cases referred following ultrasound queries had a normal brain appearance on fetal MRI and are presented as follows.

Ultrasound query CNS abnormality

1. One fetus was referred following a query of fetal unilateral right ventriculomegaly with an atrial diameter of 10.5 mm. Fetal MRI was performed 2 weeks following the ultrasound scan and showed normal measurements of the lateral ventricles (6.6 and 6 mm). The mother had a negative infection screening and there were no physical signs suggestive of a genetic syndrome at the child’s birth.
2. One fetus was referred following an ultrasound query of agenesis of the corpus callosum. Fetal brain MRI showed normal brain appearances for gestation and the corpus callosum was visualised in its entirety.
3. One fetus was referred following an ultrasound query of the presence of an interhemispheric arachnoid cyst which was not detected on fetal brain MRI.
4. One fetus was referred following a query of enlarged cavum septum pellucidum. 2D measurements of the cavum septum pellucidum on fetal and postnatal MRI were within the normal range.
5. One fetus was referred following ultrasound findings of bilateral choroid plexus cysts, distorted shape of the anterior horns, and left ventricular echogenic focus. Fetal and postnatal MRI showed normal brain appearances for GA.

Ultrasound non-CNS abnormality

1. One fetus had a right-sided suprarenal cyst. Genetic testing on amniotic fluid showed a normal karyotype. The child had a formal developmental assessment at 13.5 months of age which was within the normal range.
2. One fetus was referred following a query of diaphragmatic hernia. A diagnosis of diaphragmatic hernia was ruled out on fetal MRI, on follow-up ultrasound scans and postnatal chest X-ray. The child had a normal formal developmental assessment at 18.7 months of age.
3. One fetus was referred following a resolved cystic hygroma. There was no evidence of a cystic hygroma on fetal MRI. The child had a normal formal developmental assessment at 29.4 months of age.
4. One fetus was referred following a diagnosis of a left-sided congenital cystic adenomatoid malformation which had resolved by 38 gestational weeks as confirmed on ultrasound. A formal developmental assessment was performed at 20 months of age which was within normal limits.
5. One fetus was referred due to a prominent nuchal fold measurement of 6.5 mm. There were no physical signs suggestive of genetic syndromes at birth. The child had a formal developmental assessment at 16 months of age and an ASQ at 33 months of age, and both were within the normal range.

## Abbreviations

GA: Gestational age
CSF: Cerebrospinal fluid
MRI: Magnetic resonance imaging
CSP: Cavum septum pellucidum and vergae
ssTSE: Single shot turbo spin echo
SD: Standard deviation
